# The application of coronary CT angiography combined with dynamic electrocardiogram in the diagnosis of myocardial ischemia in coronary heart disease

**DOI:** 10.3389/fcvm.2026.1748856

**Published:** 2026-01-30

**Authors:** Zhaoyu Zhang, Dingrong Ji, Jing Wen

**Affiliations:** 1 Department of General Medicine, Weifang Community Health Care Center, Shanghai, China; 2 Department of Health Management Center, Zhongshan Hospital, Fudan University, Shanghai, China; 3 Department of General Practice, Zhongshan Hospital, Fudan University, Shanghai, China

**Keywords:** CAG, CCTA, DCG, diagnostic value, myocardial ischemia in coronary heart disease

## Abstract

**Objective:**

This study aims to analyze the diagnostic methods for myocardial ischemia in coronary heart disease and compare the diagnostic performance of coronary CT angiography (CCTA), dynamic electrocardiogram (DCG), and CCTA-DCG.

**Methods:**

This study retrospectively selected 300 patients who underwent CCTA and DCG examinations due to chest pain and suspected coronary heart disease in a hospital in Shanghai from January 2023 to June 2025. These patients were further divided into the ischemia group and the non-ischemia group based on whether they were diagnosed with myocardial ischemia in coronary heart disease. The patients' routine and clinical data were selected. Subsequently, the diagnostic performance was analyzed, and its accuracy was evaluated through the receiver operating characteristic (ROC) curve.

**Results:**

In the ischemia group, the CCTA parameters NCPV, CPV, Agaston score, and TPV were all higher than those in the non-ischemia group (*P* < 0.05). The DCG parameters, triangular index, and heart rate deceleration force in the ischemia group were lower than those in the non-ischemia group, while the LF/HF ratio was higher (*P* < 0.05). The sensitivity, specificity, positive predictive value, negative predictive value and accuracy of CCTA diagnosis were 90.66%, 70.34%, 82.50%, 83.00% and 82.67% respectively, while those of DCG diagnosis were 78.02%, 52.54%, 71.72%, 60.78% and 68.00% respectively. The combined diagnosis of CCTA-DCG was 96.15%, 91.53%, 94.59%, 93.91% and 94.33% respectively. The sensitivity, specificity, positive predictive value, negative predictive value and accuracy of CCTA-DCG diagnosis were significantly higher than those of CCTA and DCG diagnosis (*P* < 0.05). The AUC value was 0.966 (95% CI: 0.943–0.989).

**Conclusion:**

The combination of CCTA and DCG has high accuracy in diagnosing myocardial ischemia in coronary heart disease, which is of great significance for the clinical diagnosis of myocardial ischemia in coronary heart disease.

## 1 Introduction

Myocardial ischemia in coronary heart disease, as a major cardiovascular disease, has seen a continuous rise in global incidence and mortality, becoming a significant public health burden (1–3). Currently, the incidence rate of myocardial ischemia in coronary heart disease shows significant regional differences and demographic characteristics worldwide. Even in developed countries, despite active prevention and control measures, it remains high. In developing countries, it is growing rapidly due to urbanization, population aging, and the Westernization of lifestyle (4, 5). According to epidemiological data, myocardial ischemia in coronary heart disease is not only the leading cause of death and disability among adults, but also due to its high recurrence rate, high medical costs, and long-term impact on patients' quality of life (6). Myocardial ischemia in coronary heart disease not only leads to an imbalance between myocardial oxygen supply and demand, causing acute events such as angina pectoris and myocardial infarction, but also serves as the primary pathological basis for heart failure, malignant arrhythmias, and even sudden cardiac death, seriously threatening the quality of life and long-term prognosis of patients (7). Therefore, early and precise diagnosis, reliable risk stratification, and timely intervention of myocardial ischemia in coronary heart disease are crucial for improving patient outcomes and reducing the disease burden (8).

Currently, selective coronary angiography (CAG) is regarded as the “gold standard” for diagnosing coronary artery stenosis and assessing the degree of myocardial ischemia (9). However, CAG is an invasive procedure, and its inherent limitations cannot be ignored. Firstly, it is accompanied by risks such as vascular injury, bleeding, hematoma, contrast agent allergy, arrhythmia, and even coronary artery dissection or perforation (10). Secondly, patients are exposed to ionizing radiation and iodine-based contrast agents with nephrotoxicity, which poses an additional threat to those with impaired renal function (11). Thirdly, CAG mainly provides information on anatomical stenosis and has limited efficacy in assessing whether stenosis leads to functional myocardial ischemia or microvascular dysfunction (12). These risks and limitations make CAG unsuitable as a widely used screening or routine follow-up method, especially for patients with atypical symptoms, those at the borderline of risk, or those unable to tolerate invasive procedures.

Against this backdrop, the development of safe, efficient and non-invasive diagnostic strategies has become particularly urgent. Coronary computed tomography angiography (CCTA) is a non-invasive imaging technique that can be used to assess myocardial ischemia, cardiovascular status, and a series of other phenomena (13). Studies have also found that CCTA has clinical value in the diagnosis of cardiovascular diseases, suggesting that it may provide some reference for the diagnosis of myocardial ischemia in coronary heart disease (13–16). Dynamic electrocardiogram (DCG) is a non-invasive examination technique that can comprehensively record the dynamic changes of the patient's electrocardiogram signals during daily activities, providing important information for evaluating cardiac autonomic nerve function, myocardial ischemia, and arrhythmias etc. (17). Both CCTA and DCG are of great significance for the diagnosis of myocardial ischemia in coronary heart disease (18–20). Theoretically, the combination of CCTA and DCG is expected to achieve a complementary assessment from “structure” to “function”, forming a more comprehensive diagnostic perspective. In a study, it was found that when the DSE of patients with chest pain was normal, CCTA was able to detect coronary artery stenosis; CCTA: The true positive and false positive rates ratio of DSE was 1.70 (95% CI 1.65–1.75) and 1.00 (95% CI 0.91–1.09), respectively (21). This indicates that the combination of these two methods for diagnosis can provide a more accurate diagnosis for patients, and can make up for the shortcomings of the two individual diagnoses. Although there are studies exploring the value of their use alone or in combination in the diagnosis of myocardial ischemia in coronary heart disease, there are certain contradictions in the numerical accuracy of the existing studies, and the evidence for the clinical application of combined diagnosis is insufficient (22, 23). Therefore, this study employs CCTA combined with DCG monitoring to analyze its diagnostic accuracy for myocardial ischemia in coronary heart disease, aiming to provide a safer and more effective solution for the diagnosis of myocardial ischemia in coronary heart disease and offer an effective reference for clinical treatment.

## 2 Material and methods

### 2.1 Participants

Continuity selected 300 patients suspected of having coronary heart disease who had undergone CCTA, DCG and CAG examinations at hospitals in Shanghai as the research subjects. The study was approved by the Medical Ethics Committee of the hospital, with the ethics number being B2021-0771. Patients had signed the informed consent form.

Inclusion criteria: (1) Age ≥ 18 years; (2) Complete clinical data; (3) No other infectious diseases; (4) Normal liver and kidney functions; (5) No severe adverse reactions to CCTA and DCG examinations; and (6) The CCTA, DCG, and CAG examinations were successfully completed within 2 weeks of admission. Exclusion criteria: (1) Presence of major other diseases; (2) Malignant tumors; (3) Missing data; (4) Known allergies to any drugs used in this study.

Among the 300 patients, based on coronary artery examination CAG, those with a lumen stenosis degree of ≥50% and FFR ≤ 0.80 were diagnosed with myocardial ischemia in coronary heart disease (16). This resulted in the formation of the ischemia group and the non-ischemia group. Based on the examination methods used, the diagnostic techniques are classified as CCTA, DCG, and CCTA-DCG. The condition for CCTA-DCG to diagnose myocardial ischemia in coronary heart disease is that both the CCTA and DCG diagnoses are positive.

### 2.2 General and clinical information

The data from patient examinations were retrieved from a hospital in Shanghai. General information includes gender, age, BMI, smoking history, and drinking history. Clinical characteristics include New York Heart Association Functional Classification (NYHA grade), diabetes history, hypertension history, blood pressure (systolic pressure, diastolic pressure), family history of early-onset coronary heart disease (defined as male first-degree relatives <55 years old, female <65 years old diagnosed), total cholesterol, low-density lipoprotein cholesterol, high-density lipoprotein cholesterol, and triglyceride levels. Medication usage: including the proportion of use of antihypertensive drugs, and hypoglycemic drugs.

### 2.3 CCTA examination

All 300 patients suspected of having coronary heart disease combined with acute myocardial ischemia in coronary heart disease underwent coronary CT angiography using dual-source CT upon admission. Pre-examination preparations were carried out, instructing the patients to remain fasting before the examination, taking metoprolol 25–125 mg orally to control heart rate (<75 beats/min), sublingually administering nitroglycerin 0.6 mg, and performing breath-holding exercises. Using a high-pressure injector to inject iodixanol 400 mgI/mL through the median cubital vein at a rate of 5 mL/s. The image scan selected the aortic arch level as the region of interest, with a delay of 6 s, for tracking trigger, setting the threshold at 100 Hu, starting the scan from 1 cm below the carina and continuing until 2 cm below the diaphragm; scanning parameters: tube voltage 70–120 kV, tube current 350–700 mAs, matrix 1,024 × 1,024, slice thickness 0.75 mm, field of view 20 cm × 20 cm. After the examination, all coronary artery CTA images were post-processed and plaque quantification analyzed using the dedicated vascular analysis software Syngo.via VB60. The plaque segmentation was performed using a U-Net architecture segmentation model based on deep learning. This model was trained on a large-scale coronary artery CTA dataset and can automatically identify the lumen contour and the outer elastic membrane boundary. The plaque composition was distinguished according to the preset CT value threshold: non-calcified plaques were defined as vascular wall tissues with CT values ranging from 30 to 130 HU, and calcified plaques were defined as regions with CT values greater than 130 HU. The analysis process was semi-automated: initially, the software automatically completed the initial segmentation and classification, followed by independent manual correction by two experienced cardiologic imaging physicians, with a focus on verifying the consistency of plaque boundaries and component classifications. In case of disagreement, after consultation, a third senior physician would arbitrate to determine the final result. The final obtained quantitative parameters included: non-calcified plaque volume (NCPV), calcified plaque volume (CPV), Agaston score, and total plaque volume (TPV, calculated formula: TPV = NCPV + CPV).

The assessment of stenosis in all CCTA images was strictly conducted in accordance with the version 2.0 of the Coronary Artery Disease Reporting and Data System (24). In this study, the threshold for defining significant stenosis with hemodynamic significance was Coronary Artery Disease Reporting and Data System level 3 (≥50% stenosis). If the CCTA results showed that there were mixed plaques in any segment, with severe stenosis of the lumen (≥50%), then it was diagnosed as myocardial ischemia in coronary heart disease (13). The interpretation and plaque analysis of all coronary artery CCTA images were independently completed by two radiologists with over 10 years of experience in cardiac imaging diagnosis, without knowing each other's results and blinded to the CAG status of the patients. For continuous variables, the average value of the measurement results from the two physicians was taken as the final value. For categorical variables, if there was a disagreement, consensus was reached through negotiation; if negotiation failed to reach a consensus, the third senior cardiac imaging chief physician would arbitrate and make the decision.

### 2.4 Dynamic electrocardiogram monitoring

Immediately after admission, the patients were fitted with DCG monitoring equipment (MedExMECG-200, Beijing). The paper speed was controlled at 25 mm/s, and the voltage was set at 10 mm/mV. During the monitoring period, the patients were required to maintain normal daily activities. After 24 h, the equipment was retrieved, and the following indicators were recorded: average heart rate, triangular index, Low Frequency/High Frequency (LF/HF), average QT RR, and heart rate deceleration force.

If a patient experienced symptoms such as chest tightness and chest pain, the DCG observed a 1.5 mm horizontal drop in the ST segment, accompanied by an inverted T wave, and this condition persisted for approximately 5 min; it was diagnosed as myocardial ischemia in coronary heart disease (17). All the interpretation and analysis of DCG were independently completed by two radiologists with over 10 years of experience in cardiac imaging diagnosis. They were unaware of each other's results beforehand and remained blind to the CAG status of the patients. For continuous variables, the average value of the measurements by the two doctors was taken as the final value. For categorical variables, if there was a disagreement, consensus was reached through negotiation; if negotiation failed to reach a consensus, a third senior cardiologist in charge of cardiac imaging would arbitrate and make a decision.

### 2.5 Statistical analysis

The data were analyzed using SPSS 27.0.1 software. Normality tests (Shapiro–Wilk test) were conducted for all variables. Quantitative data that followed a normal distribution were expressed as $\left(\bar{x}\;\pm s\right)$, and comparisons between the two groups were performed using the *t* test; for data that did not follow a normal distribution, the median and interquartile range [*M* (Q_25_, Q_75_)] were used for presentation, and the Wilcoxon test was employed for comparisons between the two groups; Count data were expressed as cases (%) and comparisons among groups were conducted using the $\chi^{2}$ test. The condition for CCTA-DCG to diagnose myocardial ischemia in coronary heart disease is that both the CCTA and DCG diagnoses are positive. Based on the diagnostic results, the ROC curve was plotted using SPSS 27.0.1 software, and the diagnostic capabilities of CCTA, DCG, and CCTA-DCG were evaluated by calculating the area under the curve (AUC). If the AUC is greater than 0.7, it can be considered to have good diagnostic performance. *P* < 0.05 indicated statistical significance.

## 3 Results

### 3.1 Comparison of general and clinical information

There were no statistically significant differences in gender, disease duration, BMI, smoking history, alcohol consumption history of diabetes, hypertension, blood pressure (systolic and diastolic) and Family history of early-onset CHD between the two groups (*P* > 0.05). The proportion of patients in the ischemia group with NYHA ≥Ⅲ, total cholesterol, low-density lipoprotein cholesterol, high-density lipoprotein cholesterol, and triglyceride levels, Medication usage: including the proportion of use of antihypertensive drugs, and hypoglycemic drugs were higher than that in the non-ischemia group (*P* < 0.05). See Table 1.

**Table 1:** Comparison of general and clinical information.

General information	Ischemia (*n* = 182)	Non-ischemia (*n* = 118)	*P*
Gender/*n* (%)			0.531
Male	141 (77.47)	95 (80.51)	
Female	41 (22.53)	23 (19.49)	
Age, (year, $\bar{x}\;\pm s$)	60.92 ± 9.50	60.98 ± 10.88	0.917
BMI $\left(\bar{x}\;\pm s\right)$	23.65 ± 2.27	24.17 ± 2.67	0.082
Smoking history/*n*	118 (64.84)	82 (69.49)	0.306
Allergy history/*n*	129 (70.88)	87 (73.73)	0.591
NYHA/*n* (%)			0.001
≥Ⅲ	145 (79.67)	74 (62.71)	
<Ⅲ	37 (20.33)	44 (37.29)	
Systolic pressure (mmHg, $\bar{x}\;\pm s$)	125.63 ± 10.33	124.46 ± 9.42	0.327
Diastolic pressure (mmHg, $\bar{x}\;\pm s$)	75.95 ± 8.06	76.56 ± 8.07	0.509
Hypertension/*n*	98 (53.85)	63 (53.39)	0.938
Diabetes/*n*	54 (29.67)	35 (29.66)	0.999
Family history of early-onset CHD/*n*	19 (10.44)	7 (5.93)	0.175
Total cholesterol (mmol/L, $\bar{x}\;\pm s$)	4.54 ± 1.14	4.21 ± 0.83	0.005
Low-density lipoprotein cholesterol (mmol/L, $\bar{x}\;\pm s$)	2.85 ± 0.89	2.54 ± 0.78	0.003
High-density lipoprotein cholesterol (mmol/L, $\bar{x}\;\pm s$)	1.17 ± 0.30	0.81 ± 0.10	<0.001
Triglyceride (mmol/L, $\bar{x}\;\pm s$)	1.79 ± 0.46	2.15 ± 0.50	<0.001
Antihypertensive drugs/*n*	51 (28.02)	42 (35.59)	0.166
Hypoglycemic drugs/*n*	47 (25.82)	33 (27.97)	0.682

NYHA, New York Heart Association Functional Classification.

### 3.2 Comparison of CCTA parameters between the two groups of patients

**Figure 1:**
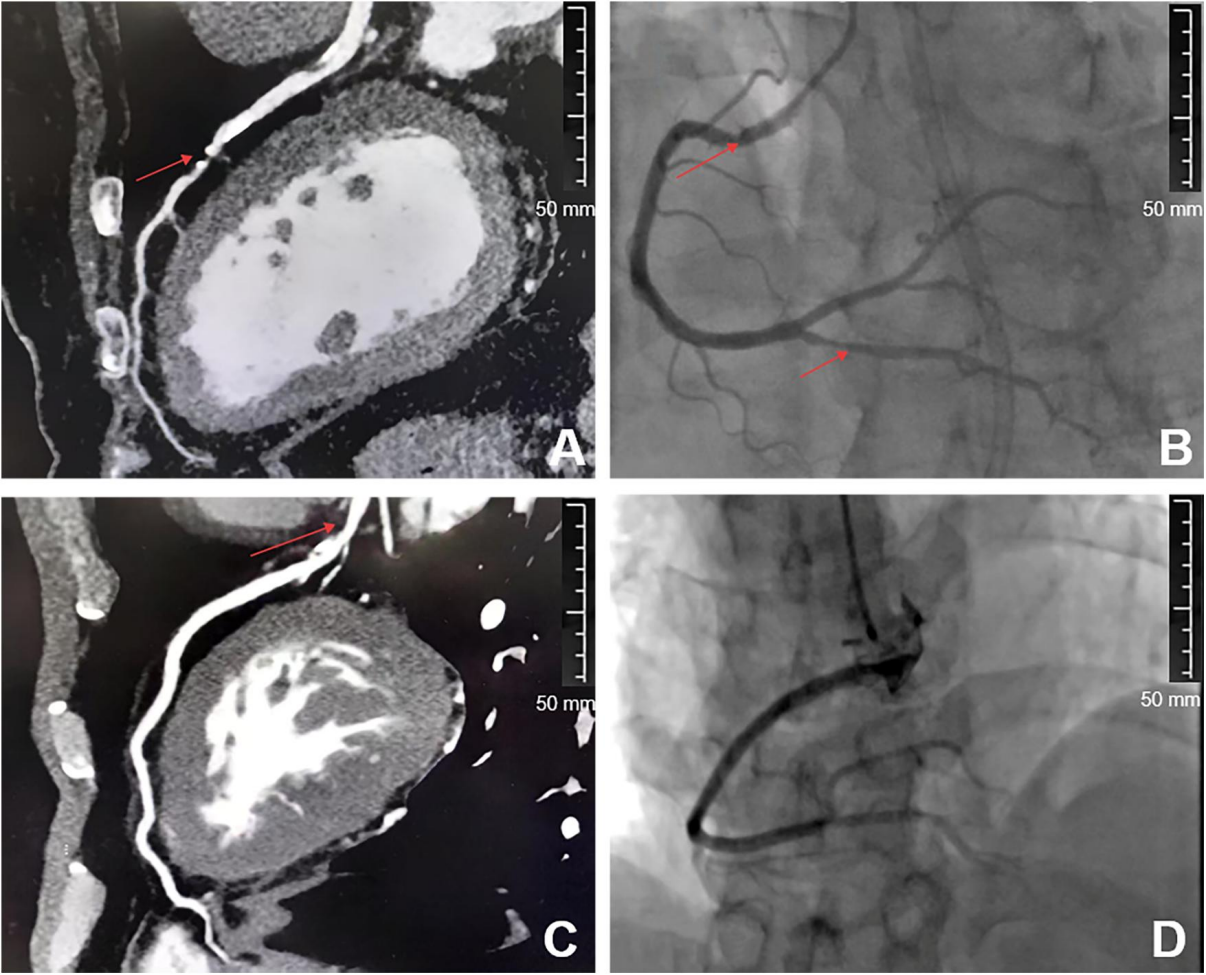
Comparison of CCTA images between the two groups of patients. **A** and **C** are CAG images, while **B** and **D** are CCTA images. **A** and **B** are patients diagnosed as positive by CCTA, while **C** and **D** are patients diagnosed as negative by CCTA. The arrows in **A** and **B** of the picture represent vascular plaques and stenosis.

The CCTA parameters NCPV, CPV, and TPV in the ischemia group were all higher than those in the non-ischemia group (*P* < 0.05), while there was no statistically significant difference in the reconstruction index and vascular volume (*P* > 0.05). See Table 2. The CCTA images showed that in the patients diagnosed with myocardial ischemia in coronary heart disease, clear atherosclerotic plaques can be observed, causing significant narrowing of the lumen. In the images of non-ischemia patients, the coronary artery walls were smooth, with no plaques or only very small and insignificant plaques, and there was no narrowing or very mild narrowing of the lumen, with normal blood flow. See Figure 1.

**Table 2:** Comparison of CCTA parameters between the two groups of patients.

Clinical data	Ischemia (*n* = 182)	Non-ischemia (*n* = 118)	*P*
NCPV (mm^3^, [*M* (Q_25_, Q_75_)])	250.37 (215.66, 281.67)	221.92 (184.36, 263.13)	<0.001
CPV (mm^3^, $\bar{x}\;\pm s$)	39.26 ± 6.81	29.76 ± 7.35	<0.001
TPV (mm^3^, $\bar{x}\;\pm s$)	251.92 ± 47.56	217.27 ± 39.93	<0.001
Vascular volume (mm^3^, [*M* (Q_25_, Q_75_)])	572.90 (556.54, 588.03)	576.89 (560.94, 592.47)	0.147
Calcification score (AU, [*M* (Q_25_, Q_75_)])	350.00 (181.00, 652.00)	116 (86.00, 225.00)	<0.001
Reconstruction Index $\left(\bar{x}\;\pm s\right)$	1.60 ± 0.50	1.60 ± 0.48	0.983

NCPV, non-calcified plaque volume; CPV, calcified plaque volume; TPV, total plaque volume.

### 3.3 Comparison of DCG indices between the two groups of patients

**Figure 2:**
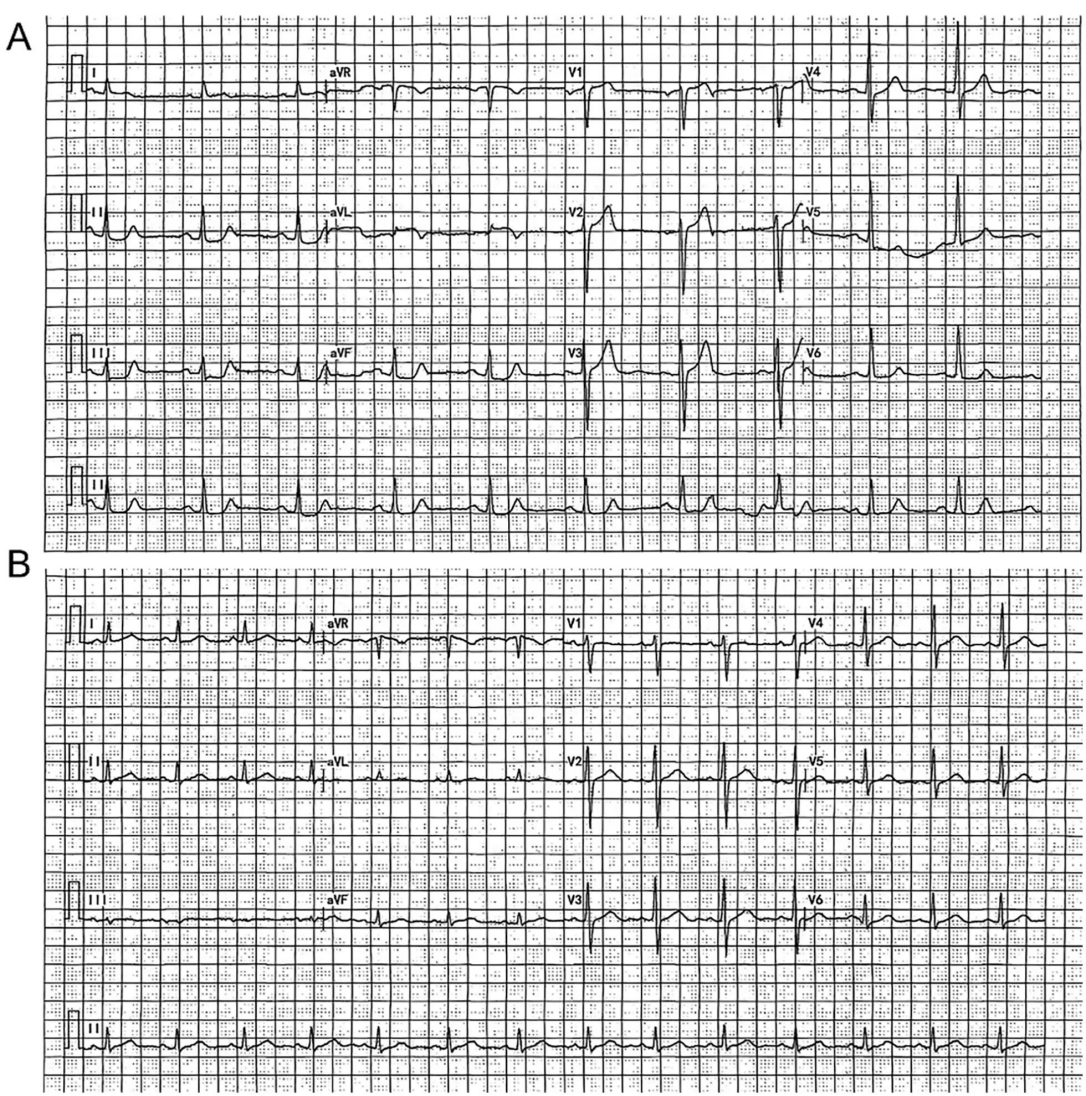
Comparison of dynamic electrocardiogram between the two groups of patients. Patient **A** was diagnosed as positive by the DCG, while patient **B** was determined as negative by the DCG.

The DCG parameters, the triangular index and the heart rate deceleration force, of the ischemia group were lower than those of the non-ischemia group, and the LF/HF ratio was higher than that of the non-ischemia group (*P* < 0.05). There was no statistically significant difference in the average QT interval and average heart rate (*P* > 0.05). See Table 3. The DCG showed that ischemia patients presented phenomena such as ST segment depression or elevation, T wave changes, and various arrhythmias, while non-ischemia patients did not have ischemic ST-T changes. See Figure 2.

**Table 3:** Comparison of DCG indices between the two groups of patients.

Clinical data	Ischemia (*n* = 182)	Non-ischemia (*n* = 118)	*P*
Average heart rate (time, $\bar{x}\;\pm s$)	73.00 ± 5.25	73.00 ± 4.40	0.368
Average QT interval (RR, ms, $\bar{x}\;\pm s$)	818.94 ± 46.20	820.29 ± 51.41	0.813
Heart rate deceleration force (ms, $\bar{x}\;\pm s$)	40.29 ± 5.14	44.33 ± 5.42	<0.001
Triangular Exponential $\left(\bar{x}\;\pm s\right)$	4.25 ± 0.47	4.84 ± 0.55	<0.001
LF/HF $\left(\bar{x}\;\pm s\right)$	2.54 ± 0.43	2.10 ± 0.53	<0.001

LF/HF, low frequency/high frequency.

### 3.4 Comparison of CCTA, DCG and combined detection methods with CAG results

CCTA examination identified 200 cases of ischemia patients and 100 cases of non-ischemia patients; DCG examination identified 198 cases of ischemia patients and 102 cases of non-ischemia patients. The combined use of CCTA and DCG detected 185 cases of ischemia patients and 115 cases of non-ischemia patients. See Table 4.

**Table 4:** Comparison of DCG, CCTA and combined detection methods with CAG results (*n*).

Index	CAG		Total
	Positive	Negative	
CCTA			
Positive	165 (55.00)	35 (11.67)	200
Negative	17 (5.67)	83 (27.67)	100
DCG			
Positive	142 (47.33)	56 (18.67)	198
Negative	40 (13.33)	62 (20.67)	102
CCTA + DCG			
Positive	175 (58.33)	10 (3.33)	185
Negative	7 (2.33)	108 (36.00)	115
Total	182	118	300

CCTA, coronary CT angiography; DCG, dynamic electrocardiogram; CAG, conventional coronary angiography.

### 3.5 Comparison of diagnostic performance of each group

The sensitivity, specificity, positive predictive value, negative predictive value and accuracy of CCTA diagnosis were 90.66%, 70.34%, 82.50%, 83.00% and 82.67% respectively, while those of DCG diagnosis were 78.02%, 52.54%, 71.72%, 60.78% and 68.00% respectively. The combined diagnosis of CCTA-DCG was 96.15%, 91.53%, 94.59%, 93.91% and 94.33% respectively. The sensitivity, specificity, positive predictive value, negative predictive value and accuracy of CCTA-DCG diagnosis were significantly higher than those of CCTA and DCG diagnosis (*P* < 0.05). See Table 5.

**Table 5:** Analysis of diagnostic performance of DCG, CCTA and combined detection methods.

Index	Sensitivity (%)	Specificity (%)	Positive predictive value (%)	Negative predictive value (%)	Accuracy (%)
CCTA	90.66 (165/182)	70.34 (83/118)	82.50 (165/200)	83.00 (83/100)	82.67 (248/300)
DCG	78.02 (142/182)	52.54 (62/118)	71.72 (142/198)	60.78 (62/102)	68.00 (204/300)
CCTA-DCG	96.15 (175/182)	91.53 (108/118)	94.59 (175/185)	93.91 (108/115)	94.33 (283/300)
*P*	<0.001	<0.001	<0.001	<0.001	<0.001

CCTA, coronary CT angiography; DCG, dynamic electrocardiogram; CAG, conventional coronary angiography.

### 3.6 ROC curve analysis

**Figure 3:**
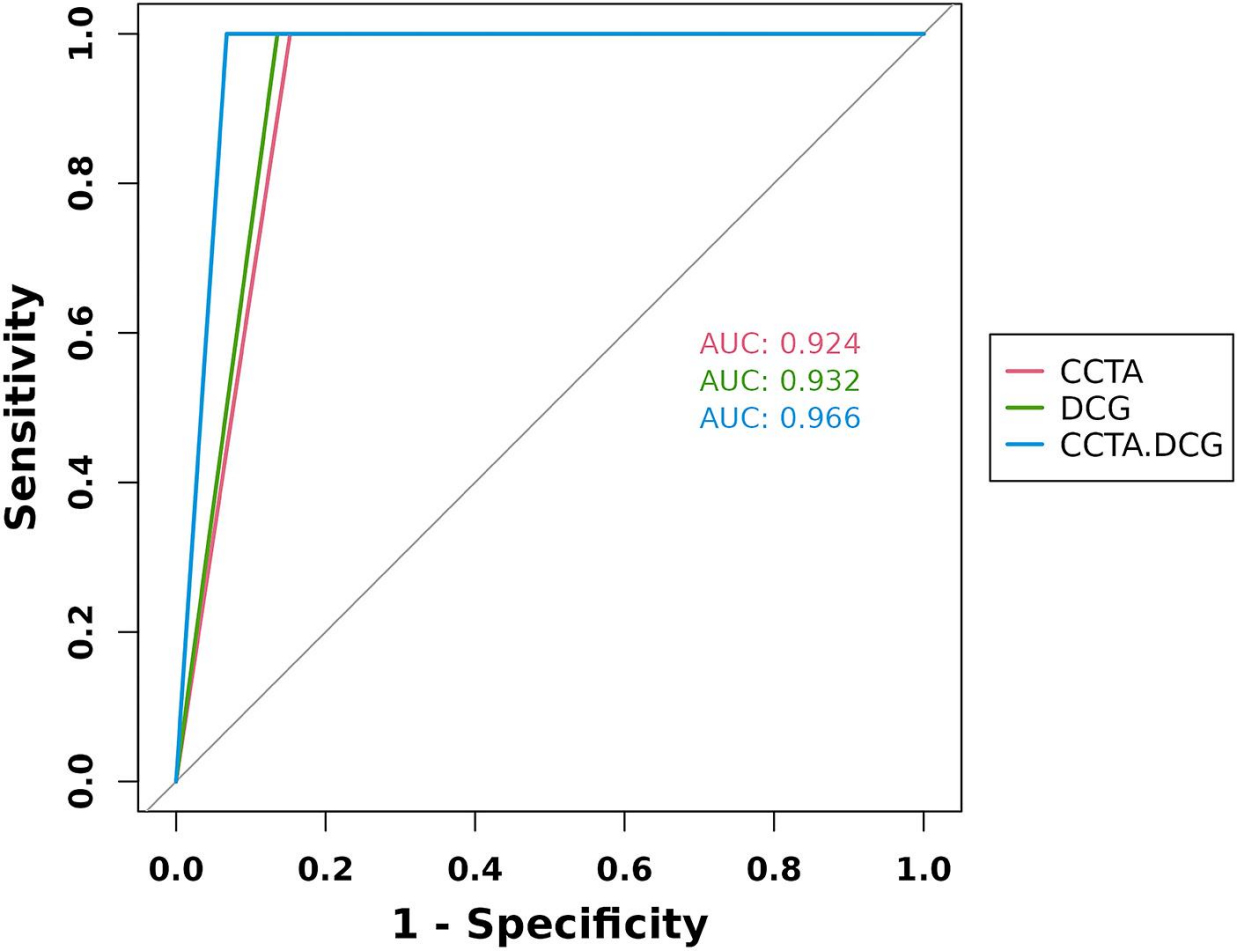
ROC curve analysis of DCG, CCTA, and combined detection methods. CCTA, coronary CT angiography; DCG, dynamic electrocardiogram; AUC, area under the curve.

The ROC curve analysis indicated that the AUC values for CCTA, DCG, and CCTA-DCG in diagnosing ischemia were 0.924 (95% CI: 0.891–0.956), 0.932 (95% CI: 0.901–0.963), and 0.966 (95% CI: 0.943–0.989), respectively. See Figure 3.

## 4 Discussion

Myocardial ischemia in coronary heart disease is a common cardiovascular disease among the elderly with a high incidence rate. Its main pathological features include coronary atherosclerosis or plaque formation, narrowing or even blockage of the lumen, which can damage the patient's cardiac contractile function and increase the risk of heart failure. Therefore, it is of great significance for clinical diagnosis and treatment to adopt scientific and objective non-invasive diagnostic methods (25–27). This study utilized both separate and combined CCTA and DCG to explore their diagnostic value. The results showed that the combination of CCTA and DCG could effectively assess myocardial ischemia in coronary heart disease accurately, providing a new and effective tool for the early diagnosis of myocardial ischemia in coronary heart disease.

Studies have shown that NYHA classification are important indicators for assessing the severity of myocardial ischemia in coronary heart disease (28). The results of this study indicated that the proportion of patients with NYHA classification of grade III or above in the ischemia group was higher than that in the non-ischemia group (*P* < 0.05). In addition, the levels of total cholesterol, low-density lipoprotein cholesterol, high-density lipoprotein cholesterol and triglycerides in the ischemia group, as well as the usage of medications (including the proportion of antihypertensive drugs and hypoglycemic drugs used), were all higher than those in the non-ischemia group (*P* < 0.05). The NYHA classification of patients with poorer conditions and the data on their high blood lipid and hypertension indicate significant abnormalities in their cardiac structure and function. These conditions are important risk factors for coronary artery disease (29). This is consistent with the results of this study, possibly because the severity of the disease itself is an important influencing factor for the occurrence and development of myocardial ischemia in coronary heart disease. As the disease progresses, the degree of coronary atherosclerosis will worsen, and the conditions of myocardial ischemia and hypoxia will also intensify, further increasing the risk of myocardial ischemia in coronary heart disease. In summary, NYHA classification are closely related to the occurrence of myocardial ischemia in coronary heart disease.

Previous studies have shown that NCPV, CPV, Agaston score, and TPV were important indicators reflecting the burden of atherosclerotic plaques in the coronary arteries (30). The larger the plaque volume, the more likely the degree of coronary artery stenosis were severe, the higher the risk of myocardial ischemia, and the higher the incidence of CHD (31). The results of this study indicated that, the NCPV, CPV, Agaston score, and TPV of the ischemia group were higher than those of the non-ischemia group (*P* < 0.05). Previous studies have pointed out that the increase in coronary artery plaque volume is closely related to the occurrence of myocardial ischemia in coronary heart disease (32). The results of this study are consistent with the literature because the formation and development of atherosclerotic plaques in the coronary arteries is the core pathological mechanism of myocardial ischemia in coronary heart disease. The increase in plaque volume reflects the progression of atherosclerotic lesions. When the plaque becomes unstable or ruptures, it will trigger thrombosis, which leads to acute coronary artery obstruction and myocardial ischemia, and subsequently causes myocardial ischemia in coronary heart disease. In conclusion, NCPV, CPV, Agaston score, and TPV were important indicators reflecting whether one has myocardial ischemia in coronary heart disease. Furthermore, although high calcium content may increase the complexity of the assessment, our study employed the latest iterative reconstruction technology and was interpreted by experienced doctors to maximize accuracy.

The quantitative parameters in DCG, LF/HF, triangular index, and heart rate deceleration force, can reflect the state of autonomic nerve function (33). Autonomic nerve dysfunction may play an important role in the pathogenesis of myocardial ischemia in coronary heart disease: Increased sympathetic nerve excitability and decreased vagus nerve excitability can lead to unstable cardiac electrical activity, increasing the risk of arrhythmia and myocardial ischemia (34). The results of this study indicate that the LF/HF ratio in the ischemia group was higher than that in the non-ischemia group, while the triangular index and heart rate deceleration force were lower in the non-ischemia group (*P* < 0.05). A study found that CHD patients have an imbalance in autonomic nerve function, manifested as enhanced sympathetic nerve activity and weakened vagus nerve activity (35), which is consistent with the results of this study showing an increase in LF/HF and a decrease in the triangular index and heart rate deceleration force. This may be due to the fact that the autonomic nervous system plays a crucial role in regulating the heart. In myocardial ischemia in coronary heart disease patients, due to factors such as myocardial ischemia and changes in cardiac structure, the autonomic nerve function may become disordered. Excitation of the sympathetic nerve leads to an increase in heart rate and myocardial contractility, thereby increasing myocardial oxygen consumption; reduced excitability of the vagus nerve weakens the protective effect on the heart, thereby jointly promoting the occurrence and development of myocardial ischemia in coronary heart disease. In conclusion, the low-frequency/high-frequency ratio, triangular index, and heart rate deceleration force are significantly abnormal in myocardial ischemia in coronary heart disease patients.

In the diagnosis of cardiovascular diseases, sensitivity, specificity and accuracy are the key indicators for evaluating the performance of diagnostic methods (36). Sensitivity is used to identify patients and prevent missed diagnoses; specificity is used to identify healthy individuals and avoid false diagnoses; accuracy comprehensively reflects the overall ability to make correct judgments (8). The sensitivity, specificity, positive predictive value, negative predictive value and accuracy of CCTA diagnosis were 90.66%, 70.34%, 82.50%, 83.00% and 82.67% respectively, while those of DCG diagnosis were 78.02%, 52.54%, 71.72%, 60.78% and 68.00% respectively. The combined diagnosis of CCTA-DCG was 96.15%, 91.53%, 94.59%, 93.91% and 94.33% respectively. The sensitivity, specificity, positive predictive value, negative predictive value and accuracy of CCTA-DCG diagnosis were significantly higher than those of CCTA and DCG diagnosis (*P* < 0.05). Compared with previous related literature, the results of this study show a certain degree of consistency with some of the literature (37). Previous literature reported that CCTA has a high sensitivity in the diagnosis of cardiovascular diseases (38), which is consistent with the result of 90.66% sensitivity of CCTA in this study; in addition, some literature pointed out that the specificity of CCTA varies greatly among different studies (39), and the specificity of CCTA in this study was 70.34%, which is somewhat similar to the values reported in some literature. Regarding DCG diagnosis, some literature indicated that its diagnostic performance is affected by multiple factors (40), and the sensitivity, specificity, positive predictive value, negative predictive value and accuracy of DCG in this study were relatively low, which is consistent with the view in some literature that DCG has limited diagnostic effect in certain situations. However, the combined diagnosis of CCTA-DCG demonstrated significantly better performance than a single diagnostic method in this study. Currently, there are relatively few relevant literature on the advantages of combined diagnosis, but the results of this study provide strong support for the application of combined diagnosis in the diagnosis of cardiovascular diseases. In summary, in the diagnosis of cardiovascular diseases, CCTA has certain diagnostic value, especially in terms of sensitivity, but its specificity still needs to be further improved; the diagnostic performance of DCG is relatively limited and may be affected by various factors. However, the combined diagnosis of CCTA and DCG can fully utilize the advantages of these two diagnostic methods, significantly improving the sensitivity, specificity and accuracy of the diagnosis.

In the field of medical diagnosis, ROC analysis is an important tool for evaluating the performance of diagnostic tests. AUC is a key indicator in ROC curve analysis, reflecting the ability of the diagnostic method to distinguish between disease states (41). For the diagnosis of myocardial ischemia in coronary heart disease, accurately assessing the AUC values of different diagnostic methods helps to select the most suitable diagnostic strategy, thereby improving the early diagnosis rate and treatment effect of the disease (42). The ROC results showed that the AUC value for CCTA diagnosis was 0.924 (95% confidence interval: 0.891–0.956); the AUC value for DCG diagnosis was 0.932 (95% confidence interval: 0.901–0.963); and the AUC value for the combined diagnosis of CCTA-DCG was as high as 0.966 (95% confidence interval: 0.943–0.989). This indicates that all three diagnostic methods have high diagnostic. Reference reported that CCTA and DCG have relatively high AUC values in diagnosing coronary heart disease-related diseases (21, 28), which is consistent with the AUC results of CCTA in this study. However, there are relatively few relevant literature on the combined diagnosis of CCTA-DCG, but the combined diagnosis AUC value in this study is as high as 0.966, indicating a trend of superiority over single diagnostic methods, which is consistent with some theoretical viewpoints that combined diagnosis can improve diagnostic accuracy (43). Our results showed that through ROC analysis, the diagnostic performance of DCG was higher than that of CCTA. However, the data in this article indicated that the diagnostic accuracy of CCTA was higher than that of DCG. These two do not contradict each other. CCTA is more accurate in diagnosing individuals below the current threshold. Its accuracy rate (82.7%) and specificity (83%) are similar to those of the current CCTA study (accuracy rate: 86%, specificity: 85%) (32). AUC represents the comprehensive ranking ability under all possible thresholds, and the discriminative ability of the DCG model itself may be slightly better than that of CCTA. In conclusion, the combined diagnosis of CCTA-DCG demonstrates more significant advantages. Its AUC value is the highest, indicating that the combined diagnosis can fully leverage the strengths of both diagnostic methods and further enhance the accuracy of diagnosis. This provides an important basis for choosing the more optimal myocardial ischemia in coronary heart disease diagnostic strategy in clinical practice.

However, this article still has certain limitations. The sample size is insufficient. In the future, the sample size will be expanded for further research. All the patients in this study came from the same hospital, which may have regional limitations. In the future, a prospective and multi-center approach will be adopted to further verify it.

In conclusion, in the diagnosis of cardiovascular diseases, CCTA has certain diagnostic value, especially in terms of sensitivity, but its specificity needs to be further improved. The diagnostic accuracy of DCG is relatively limited and may be affected by various factors. However, the combined diagnosis of CCTA-DCG can fully leverage the advantages of both diagnostic methods, significantly enhancing the sensitivity, specificity and accuracy of the diagnosis. It is expected to become a non-invasive alternative to CAG diagnosis, providing a more reliable means for the precise diagnosis of cardiovascular diseases. At the same time, the results of this study also suggest the myocardial ischemia in coronary heart disease when evaluating and comparing diagnostic methods, various factors such as the characteristics of the research subjects and the research methods need to be fully considered to ensure the accuracy and reliability of the research results, providing more valuable references for clinical practice.

## Data Availability

The raw data supporting the conclusions of this article will be made available by the authors, without undue reservation.
